# The importance of airway and lung microbiome in the critically ill

**DOI:** 10.1186/s13054-020-03219-4

**Published:** 2020-08-31

**Authors:** Ignacio Martin-Loeches, Robert Dickson, Antoni Torres, Håkan Hanberger, Jeffrey Lipman, Massimo Antonelli, Gennaro de Pascale, Fernando Bozza, Jean Louis Vincent, Srinivas Murthy, Michael Bauer, John Marshall, Catia Cilloniz, Lieuwe D. Bos

**Affiliations:** 1 Department of Intensive Care Medicine, Multidisciplinary Intensive Care Research Organization (MICRO), St James Hospital, Dublin 8., Ireland; 2 Department of Respiratory Medicine, Hospital Clinic, IDIBAPS, CIBERes, Barcelona, Spain; 3 Trinity College, Dublin, Ireland; 4 Division of Pulmonary and Critical Care Medicine, Department of Internal Medicine, University of Michigan, Ann Arbor, USA; 5 Department of Microbiology and Immunology, University of Michigan, Ann Arbor, USA; 6 Michigan Center for Integrative Research in Critical Care, Ann Arbor, MI USA; 7 Deparment of Pneumology, Institut Clinic del Tórax, Hospital Clinic of Barcelona - Institut d’Investigacions Biomèdiques August Pi i Sunyer (IDIBAPS), University of Barcelona (UB) - SGR 911- Ciber de Enfermedades Respiratorias (Ciberes), Barcelona, Spain; 8 Department of Infectious Diseases, Linköping University, Linköping, Sweden; 9 Department of Clinical and Experimental Medicine, Linköping University, Linköping, Sweden; 10 The University of Queensland, Brisbane, Australia; 11 Scientific Consultant, Nimes University Hospital, University of Montpellier, Nimes, France; 12 Department of Anesthesiology, Intensive Care and Emergency Medicine, Fondazione Policlinico Universitario A. Gemelli IRCCS, Rome, Italy; 13 Università Cattolica del Sacro Cuore, Rome, Italy; 14 National Institute of Infectious Diseases Evandro Chagas, Oswaldo Cruz Foundation, Fiocruz, Rio de Janeiro, Brazil; 15 Department of Intensive Care, Erasme University Hospital, Université Libre de Bruxelles, Brussels, Belgium; 16 University of British Columbia, Vancouver, BC V6H 3V4 Canada; 17 Department of Anesthesiology and Intensive Care Medicine, Jena University Hospital, Am Klinikum 1, 07747 Jena, Germany; 18 The Keenan Research Centre for Biomedical Science, The Li Ka Shing Knowledge Institute, St Michael’s Hospital, University of Toronto, Toronto, Ontario Canada; 19 Department of Respiratory Medicine, Infection and Immunity, Amsterdam University Medical Center, AMC, Amsterdam, The Netherlands

**Keywords:** Pneumonia, Microbiome, Infection, Ventilator-associated pneumonia, Ventilator-associated tracheobronchitis

## Abstract

During critical illness, there are a multitude of forces such as antibiotic use, mechanical ventilation, diet changes and inflammatory responses that could bring the microbiome out of balance. This so-called dysbiosis of the microbiome seems to be involved in immunological responses and may influence outcomes even in individuals who are not as vulnerable as a critically ill ICU population. It is therefore probable that dysbiosis of the microbiome is a consequence of critical illness and may, subsequently, shape an inadequate response to these circumstances.

Bronchoscopic studies have revealed that the carina represents the densest site of bacterial DNA along healthy airways, with a tapering density with further bifurcations. This likely reflects the influence of micro-aspiration as the primary route of microbial immigration in healthy adults. Though bacterial DNA density grows extremely sparse at smaller airways, bacterial signal is still consistently detectable in bronchoalveolar lavage fluid, likely reflecting the fact that lavage via a wedged bronchoscope samples an enormous surface area of small airways and alveoli. The dogma of lung sterility also violated numerous observations that long predated culture-independent microbiology.

The body’s resident microbial consortia (gut and/or respiratory microbiota) affect normal host inflammatory and immune response mechanisms. Disruptions in these host-pathogen interactions have been associated with infection and altered innate immunity.

In this narrative review, we will focus on the rationale and current evidence for a pathogenic role of the lung microbiome in the exacerbation of complications of critical illness, such as acute respiratory distress syndrome and ventilator-associated pneumonia.

## Introduction

The normal microbiota is the ecological communities of commensal, symbiotic and pathogenic microorganisms whilst the microbiome comprises all of the genetic material within a microbiota (the entire collection of microorganisms in a specific niche, such as the human gut). This can also be referred to as the metagenome of the microbiota [1, 2]. Approximately 100 billion microorganisms are found in the body due to recent discoveries in molecular analysis such as next-generation sequencing (NGS) and whole metagenome shotgun sequencing (WMGS); there is an increasing body of evidence pointing towards the dysbiosis that is often defined as an ‘imbalance’ in the microbial community that is associated with disease [3–5].

A microbiome is shaped by multiple factors including the resident flora of the animate or inanimate vicinity and the external forces that modulate this flora [6]. It becomes a changeable reflection of diversity, and so its study can provide valuable insights into the factors that drive that diversity [7]. Just as the study of global climate or the roots of language requires input from around the world, so the interpretation of the microbiome of an individual or a group of patients needs comprehensive comparative data to generate insight [8, 9]. The variability of the host microbiome—either in an individual patient over time in response to the pressures of illness [10] or in a geographically localized population in response to environmental—can yield important insight into factors that can be manipulated to improve clinical outcomes. Such factors include risk of infection, emergence of resistance, spread from the environment, host susceptibility and even the resilience of the health care system [11].

In this narrative review, we will focus on the rationale and current evidence for a pathogenic role of the lung microbiome in the exacerbation of complications of critical illness, such as acute respiratory distress syndrome (ARDS) and ventilator-associated pneumonia (VAP).

## Is the lung sterile or not?

Though for years textbooks taught that ‘the normal lung is free from bacteria’, this dogma was generally repeated without citation or argument [12]. In retrospect, this claim of lung sterility was remarkable: virtually no environment on earth exists that is so extreme in temperature, pH, salinity or nutrient scarcity that microbial communities cannot be detected [13]. Yet for more than a century, it was taken as fact that the warm, wet mucosa of the lower respiratory tract—mere inches below the microbial reservoir of the pharynx—is an exception to this rule [14–18].

Each individual has a unique microbiota profile that plays many specific functions in host nutrient metabolism, maintenance of structural integrity and protection against pathogens. There is not a unique optimal microbiota composition as it can be different for each individual [19, 20]. Thus, the ‘revolution’ in culture-independent microbiology has merely confirmed with certainty what has long been inferred indirectly: human lungs are constantly exposed to environmental bacteria. To date, more than 30 studies have used sensitive, culture-independent techniques to study lung bacteria in healthy volunteers, and none has failed to detect a distinct bacterial signal [21]. The viability of bacteria in healthy lungs has been confirmed via advanced cultivation [22] and indirectly validated via correlation with healthy alveolar immune tone in humans and mice [23, 24].

Some of the confusion regarding the existence of lung microbiota reflects flawed parallels with the lower gut microbiome, which represents a wholly different ecosystem with radically different ecologic forces. Whereas the gut lumen is densely populated by dense communities’ bacteria, lung microbiota is scarce and associated with mucosal surfaces. Whereas gut communities are relatively stable day-to-day, reflecting stable selective pressure on resident bacteria, lung communities are in constant turnover, with their identities and burdened determined by the relative balance of immigration (via microaspiration and mucosal dispersion) and elimination (via cough and mucociliary clearance). Whereas the gut microbiome is nutrient-rich and characterized by intense metabolic competition amongst dense communities, the lung microenvironment is nutrient-poor, and the primary competition is between immigrating pharyngeal microbes and locally calibrated alveolar and airway host defences attempting to minimize their outgrowth [24, 25]. These ecologic differences between the lower gut and the lungs erode somewhat in conditions of acute and chronic disease: the influx of mucus and protein-rich oedema provide nutrient sources for bacteria, and once-transient bacteria become resident, shaped by selective pressure.

Further confusion arose via misinterpretation of clinical culture protocols, which have been optimized for detection of respiratory pathogens, not the ‘background’ microbiota of uninfected patients. Sequencing-based studies have revealed that the normal microbiota of healthy lungs closely resembles that of the oropharynx [26–28] and, whilst commonly cultured, are routinely dismissed by clinical microbiology laboratories as ‘normal oral flora’.

Bronchoscopic studies have revealed that the carina represents the densest site of bacterial DNA along healthy airways, with a tapering density with further bifurcations [28]. This likely reflects the influence of micro-aspiration as the primary route of microbial immigration in healthy adults. Though bacterial DNA density grows extremely sparse at smaller airways, bacterial signal is still consistently detectable in bronchoalveolar lavage fluid, likely reflecting the fact that lavage via a wedged bronchoscope samples an enormous surface area of small airways and alveoli. Bacterial communities within the lungs of healthy volunteers are relatively homogenous; the bacteria of a given individual’s right middle lobe far more closely those of the same individual’s left upper lobe than do other individuals’ right middle lobe (i.e. intraindividual similarity is greater than interindividual similarity) [27].

## How to study the lung microbiome?

High densities of bacteria are always present on the skin, in the mouth, and in the upper respiratory tract. For this reason, it is important to avoid contamination with commensal bacteria from other sites when taking samples for investigation of the lower respiratory tract microbiome [29, 30]. Since samples from the lower respiratory tract may have a low biomass, it increases the risk for contamination that can occur at any time from sampling to sequencing [31, 32].

The first molecular techniques used for studying the bacterial microbiome in humans were based on 16S rRNA gene sequencing many years ago which is an appropriate method to assess diversity on taxonomic levels above species level. A limitation of 16S rRNA gene sequencing is that whilst bacteria can normally be identified on genus and family level, species identification usually requires simultaneous evaluation of several genes [33–35]. Newer technology of whole genome sequencing and metagenomics has shown better definition of the gut microbiome and what has currently been shown of the lung microbiome will also be significantly updated by these newer sequencing technologies [36–38].

An important matter is that when studying the lung microbiome, the pathogens and host response needs to be simultaneously studied by molecular methods, for instance, microbial metagenomics and transcriptomics. Langelier et al. [39] performed in almost 100 patients with acute respiratory failure (ARF) metagenomic next-generation sequencing (mNGS) on endotracheal aspirates (ETA) and simultaneously assessed pathogens, the airway microbiome and the host transcriptome. This study found that a single streamlined protocol offering an integrated genomic portrait of pathogen, microbiome and host transcriptome represents a new tool for diagnosis in lower respiratory tract infections (LRTI).

The progress in molecular microbiology has developed very fast in the last years and several rapid technologies will provide biological signals taking into account the interaction of the host (e.g. via digital enzyme-linked immunosorbent assay (ELISA) [40]) and the microbes (e.g. via nanorod-PCR [41]). Another technology is microgas chromatography for the analysis of bacterial function and virulence and metabolic indices of the host response on exhaled breath [42, 43].

The field of lung microbiome is no longer limited by the speed of sequencing, processing, or measurement, but rather our ability to make sense of the high-dimensional data we generate.

## Lung microbiome in ARDS

ARDS is a complication of critical illness characterized by protein-rich pulmonary oedema, hypoxaemia and alveolar inflammation. Alveolar inflammation, damage and subsequent oedema may be initiated by a change in pulmonary microbiome, or a change in lung microbiome may be initiated by an alveolar nutrient available after the onset of oedema [2]. Even though ARDS is traditionally not considered to be related to microbial changes in the lung, these physiological considerations resulted in the hypothesis that pathogenic bacteria may be present in the lung of patients with ARDS.

Kyo et al. [44] analysed the lung microbiome from the bronchoalveolar lavage fluid (BALF) of patients with ARDS found that lung bacterial burden (16S rRNA gene copy numbers tended to be increased) tended to be increased, and the alpha diversity (copy numbers and relative abundance of betaproteobacteria) was significantly decreased in ARDS patients.

In an experimental mouse model of lung injury following abdominal sepsis induced by cecal ligation and puncture, the lung microbiome was enriched with gut bacteria [3]. How did these bacteria get there? It is hypothesized that bacteria can translocate from the gut into the lymphatic system and portal circulation during critical illness [4]. If so, these changes should also be observed in patients on the ICU. Indeed, enrichment of gut bacteria was also observed in BALF from ARDS patients [3]. Gut bacteria and more specifically Enterobacterieae enrichment in patients with ARDS were confirmed in a second observational cohort study [5]. Both studies were performed in a selective cohort of patients with potential biases of prolonged antibiotic exposure before measurement. In a more recent study conducted in Europe, patients who were treated with selective decontamination of the digestive tract (SDD) during admission at the ICU, but were not treated with antibiotics prior to ICU admission, validated the specific enrichment of Enterobacterieae in the lungs of ARDS patients [45].

**Fig. 1:**
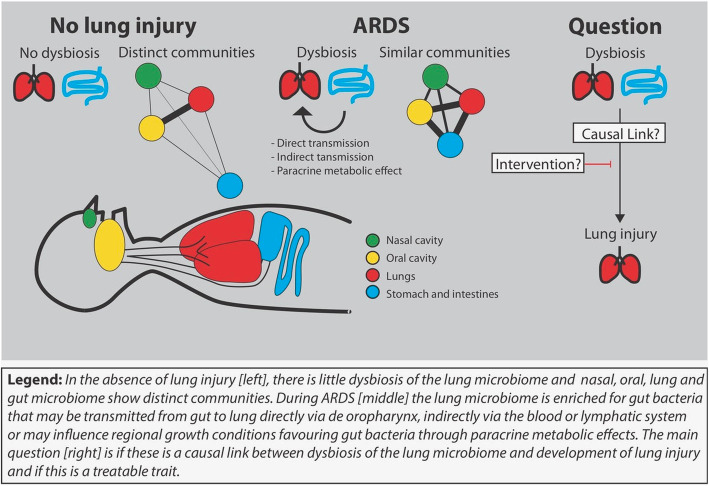
Algorithm of dysbiosis pathways in patients with pneumonia, ARDS and influence of mechanical ventilation. ARDS, acute respiratory distress syndrome

Taken together, the current body of evidence suggests that amplification of Enterobacterieae in the lung is strongly associated with ARDS. This association is not sufficiently explained by potential confounders such as geographical location of sampling, exposure to antibiotic therapy, amplification protocols or exact definitions of ARDS. The evidence for consistent dysbiosis in lung microbiome is actually stronger for ARDS than for most other respiratory diseases, where other microbes are enriched in different studies. However, no causal link between dysbiosis of the lung microbiome and development of lung injury has been established. This link needs to be further explored before we can conclude that lung microbiome dysbiosis is a potential target for treatment (Fig. 1).

## Lung microbiome during nosocomial LRTI

In ecological terms, pneumonia can be described as the collapse of local microbiome diversity and the emergence of a dominant pathogen [46]. Several studies have therefore hypothesized that the lung changes considerably during nosocomial lower respiratory tract infections. Some critically ill patients can develop pneumonia due to their clinical condition such as patients with ischaemic stroke and/or with loss of neurological control of the respiratory system. These clinical conditions can be associated to reduced airway clearance and increased bacterial translocation and therefore can develop more often respiratory infections [47]. So, the more appropriate question is ‘do patients that develop pneumonia have more dysbiosis of the lung microbiome than mechanically ventilated ICU patients who do not develop pneumonia’? Two studies addressed this problem. The first included consecutive patients at risk for pneumonia with a duration of mechanical ventilation of more than 7 days [48]. Endotracheal aspirates were performed every third day and the microbial composition was evaluated with 16S sequencing. There was a small, but significant increase in the change in beta-diversity (change in diversity of species from one environment to another) in patients who went on to develop pneumonia as compared to patients who did not develop any signs of infection and were not colonized by any bacteria according to traditional bacterial cultures. The composition of the microbiome in these patients also showed a slight enrichment of Pseudomonadales. A second study conducted had a similar design and showed no difference in the change of microbiome during mechanical ventilation between patients who did and did not develop pneumonia [49]. As discussed in the accompanying editorial, the results from these studies have elegantly shown that it is time to let go of any simplistic view of VAP pathogenesis [10]. One conclusion might be that LRTI cannot simply be defined as a collapse of bacterial ecology as this is present also in part of the patients without pneumonia who do not show any signs of pneumonia. One could also argue that the studies did not sample the alveolar space and additional studies with BALF are needed to confirm or discard these findings. Furthermore, evaluation of microbial composition may be more useful in establishing the presence of a pathogen in patients who already have a clinical suspicion of pneumonia. Indeed, with pre-test probability, metagenomics may provide valuable information on the pathogen causing pneumonia [11]. Future studies have to consider these possibilities before we disregard the lung microbiome in nosocomial pneumonia.

## Gut-lung axis and the possible interventions

In the critically ill, changes in the microbiome in all habitats, including the lungs, are particularly striking. Due to the devastating consequences of untreated severe infections, broad eradication is accepted as lesser evil and collateral damage on beneficial or commensal microbes is generally accepted. However, the potential long-term consequences of unwarranted side effects on the microbiome warrant a reassessment of the microbiome as a diagnostic or even therapeutic target. For example, dysbiosis of the gut microbiome itself has been described as a predictive factor for late-onset neonatal sepsis [50] suggesting that the microbiome can serve at least as a biomarker to predict ensuing nosocomial infection. Moreover, albeit solid data are still missing to support interventions to restore a healthy microbiome, the strategy holds promise to impact on incidence and outcome of nosocomial infection and ensuing organ injury, including ARDS [51, 52]. In the light of a better understanding of off-target effects of broad-spectrum antibiotics on the microbiome, the liberal administration of antibiotics must be discussed against more sophisticated interventions to treat the bacterial infection (non-antibiotic therapies such as bacteriophages) or manipulation of the microbiome to make the residing communities more resilient (for example probiotics). In particular, the need to combine multiple anti-infective compounds in the light of diagnostic uncertainty might outweigh the benefit of early source control and explain controversial results for aggressive antibiotic strategies. For instance, in a before-and-after study Hranjec et al. reported that the subgroup with least benefit from ‘calculated’ broad-spectrum antibiotics were patients presenting with septic shock, i.e. those in which the current paradigm would expect the highest need to initiate early anti-infective therapy [53]. Thus, a holistic approach taking the microbiome into consideration carries the potential to initiate a paradigm shift in the treatment of infections in the ICU.

**Fig. 2:**
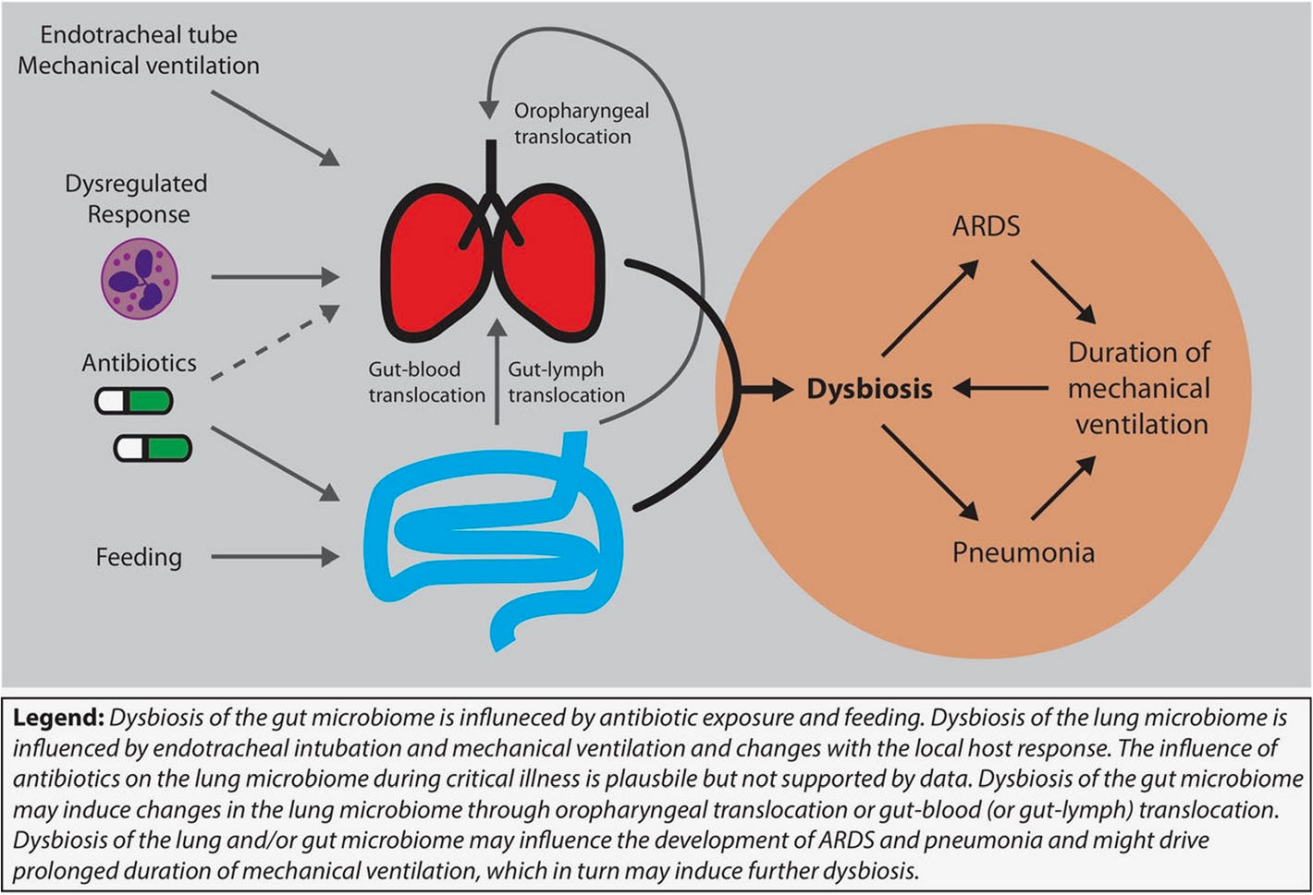
Island model for the development of lung injury based on sites of dysbiosis

As discussed in the previous paragraphs, the lung dysbiosis seems to be common in the ICU and enrichment of gut bacteria might be an important contributor to the development of lung injury and infection (Fig. 2). The relationship between gut and lung microbiome is described as the gut-lung axis [54]. Because the gut microbiome can be targeted directly or indirectly with therapeutic interventions, this is an area of active study. Investigations have thus far fallen into two specific pathways—first, using probiotics to help restore a pre-morbid microbiome, or second, to use antibiotics through an SDD approach to target specific families of organisms so as to alter the microbiome in possibly beneficial ways. Further novel pharmacologic options that have direct gut microbiome modifying effects are also under development, including faecal transplantation as a possible novel treatment for microbiota dysregulation (considering the immune system during faecal microbiota transplantation for *Clostridioides difficile* infection [55] and for the decolonization of antibiotic-resistant bacteria in the gut [56]). One of the major challenges of studying the effect of these interventions is the huge variability in the gut microbiome of critically ill patients, even during the first days of ICU admission [57]. Furthermore, any beneficial effect of these interventions on the microbiome has yet to be assessed formally in a prospective, large-scale, randomized manner. Attempting to attribute a causal impact of microbiome modifications upon clinical outcomes has been difficult to tease out as to whether changes in the microbiome are merely surrogates of some other mechanistic pathway that leads to improved clinical outcomes [58].

### Probiotics

Fundamentally, probiotics in critical illness aim to provide bacteria that may have been eradicated during the pre- and early phases of critical illness [59]. This eradication may be through administering antibiotics early in critical illness, which have been shown to greatly modify the gut microbiome [60]. Alternatively, the mere onset of critical illness—be it sepsis, ARDS or any number of conditions, is associated with alterations of the gut microbiome, which may be independent of antibiotic administration [61]. Regardless, the stated goal of probiotic administration is to restore a pre-morbid microbiome—primarily to the gut, but partially to other microbiome communities through generalized cross-talk [62]. Through yet-unknown mechanisms, administering *Lactobacillus* or *Bifidobacterium* species through a probiotic may increase the diversity of microbial species in the gut, although more studies with rigorous outcome determinations are required [63]. In the critically ill, randomized studies and meta-analyses of randomized trials demonstrate a possible benefit of probiotic administration on the outcome of ventilator-associated pneumonia, without a difference in mortality [64, 65], with a major challenge being a lack of standardization in dosing and composition of probiotic products [66]. Larger scale studies are nearing completion and further data on the impact of microbiome modifications are forthcoming in the years ahead [59].

### Selective digestive decontamination

Selective digestive decontamination, a regimen of prophylactic antibiotic administration, has been shown in small series to result in important alterations in gut microbiota, when compared with controls [67]. These changes are typically related to increasing selection for resistant organisms and decreased microbiome diversity, per a number of different metrics. Given a possible benefit on patient mortality in some randomized trials [68, 69], exploring the specific impact of this strategy on the microbiome, and related clinical outcomes, is a vital area for further study. Additionally, given burgeoning evidence of crosstalk between the lung and gut microbial communities, the impact of either of these strategies on the non-gut microbiome communities in the critically ill patient remains under-investigated.

Given the apparent conflicting goals of SDD and probiotic administration in the critically ill as it relates to the microbiome, the role of co-administration may be difficult to conceive. However, most currently used SDD regimens are unlikely to affect the administered probiotic agent, and this may be a strategy for further investigation in targeted patients [70]. Both SDD and probiotics appear to mediate their effect on patient-related outcomes through reducing the incidence of ventilator-associated pneumonia, speaking to a crucially under-investigated relationship between the two microbiome communities and host immunology, a tantalizing area for future research.

### Other treatments

Novel pharmacologic agents have also been suggested as modifiers for the gut microbiome but have yet to be formally tested in the critically ill. Butyrate, a large bowel microbial fermentation product, is being investigated in pre-clinical trials as a specific modifier of gut-derived regulatory T cells [71]. Administering a sialic acid analogue is being investigated as to whether it may reduce the burden of antibiotic-associated pathogens such as *C. difficile* by altering metabolic pathways [72]. Older drugs such as metformin may have a role, with their demonstrated effects on altering the gut microbiome in patients with diabetes [73].

The lung microbiome is clearly more difficult to target than the gut microbiome due to the lack of routine administration of bacteria and bacterial products into the airways. The low biomass environment may also cause the lung microbiome to be more prone to infection induced by the introduction of, for example, probiotics. Therefore, direct intervention in the lung microbiome may be sought via the alteration of regional growth conditions via the availability of nutrients or through immunomodulation. An example is the administration of macrolides in chronic obstructive pulmonary disease (COPD): there is a selection for anti-inflammatory microbial metabolites and an alteration of the lung microbiome [74].

All of these possible interventions speak to the importance of achieving a better understanding of the gut-lung axis in critical illness. As this understanding evolves, the possibility of personalizing interventions for individual microbiome communities, or widespread initiation of interventions such as SDD or probiotics, would be possible.

## The need for a network to support activities

Whilst patient-to-patient or staff-to-patient transmission of infection occurs within the intensive care unit, most nosocomial infections in critically ill patients arise through the invasion of normal host defences by bacteria and fungi that have become a part of an altered microbiome—either by changes in numbers or by the incorporation of species from the environment [75]. The hospital environment itself acquires a microbiome that reflects the patients that have been in it, and environmental reservoirs such as sinks, plumbing, work surfaces, and equipment can become reservoirs of resistant organisms that can infect the critically ill [76].

The inherent variability of the microbiome, therefore, provides an opportunity to study not only the individual patient, but also the forces in the environment that shape patient’s outcome, and to identify specific opportunities where the persistence and transmission of pathogens can be prevented or minimized. Because of the high prevalence of nosocomial infection, the environmental concentration of causative pathogens and the multiple risk factors for exposure, the ICU provides a unique opportunity for intensive study of the microbiome and its role in the establishment and transmission of resistant organisms. With the emergence of new models of global acute care research collaboration through the International Forum for Acute Care Trialists (InFACT; www.InFACTglobal.org), and the launch of an InFACT initiative to leverage ICU data to understand variability in patterns of resistance through the Antimicrobial Resistance in Intensive Care (AMRIC) initiative.

## Conclusion

In previous years, we believed that the normal lung was free from bacteria. Certainly, some features in the respiratory tract such as temperature, pH and nutrients were not beneficial for microbial growth. During critical illness, antibiotic use, mechanical ventilation, diet changes and inflammatory responses can bring the microbiome to dysbiosis.

With the use of molecular techniques, we have had the opportunity to study the lung microbiome and not only in the microbial aspect but also in the responses from the host. One of the most important aspects to better determine the physiopathology of host-pathogen interaction in pulmonary complications such as ARDS and VA-LRTI is the gut-lung axis. Further study of patients with disease in the respiratory tract will help us to better determine microbial diversity and constitution when comparing healthy and diseased subjects.

Dysbiosis and analysis of extra-pulmonary microbiome have helped to understand the complex interaction of bacterial clearance in the lung tissue and the off-target effects of broad-spectrum antibiotics on the microbiome.

Through therapies targeting host-pathogen interaction and the development of advance molecular testing, we will be able to have a deeper understanding in the analysis of the lung microbiome.

## Data Availability

Attached to the manuscript.
